# Molecular Identification of *Schistosoma* Species Associated with Atypical Urinary Eggs in Abuja (Nigeria): Evidence of Potential Zoonotic Transmission

**DOI:** 10.3390/tropicalmed11060170

**Published:** 2026-06-22

**Authors:** Solomon Monday Jacob, Sophie Y. Akinbo, Oluwaremilekun G. Ajakaye, Uwem F. Ekpo, Zainab Omoruyi, Temitope Agbana, Louise Makau-Barasa, Moses O. Aderogba, Jan-Carel Diehl, David Bell, Adedotun A. Bayegun, Michael A. Okungbowa, Juliana A-Enegela, Frederick O. Akinbo

**Affiliations:** 1 Department of Medical Laboratory Science, University of Benin, Benin 300213, Nigeria; zainab.omoruyi@uniben.edu (Z.O.);michael.okungbowa@uniben.edu (M.A.O.); fredrick.akinbo@uniben.edu (F.O.A.); 2 Department of Public Health, Federal Ministry of Health, Abuja 904101, Nigeria; 3 Department of Molecular Biology Diagnosis, Northwestern Memorial Hospital, Chicago, IL 60611, USA; sophieakinbo@gmail.com; 4 Laboratory of Molecular Parasitology and Genomics of Neglected Tropical Diseases, Department of Animal and Environmental Biology, Adekunle Ajasin University, Akungba-Akoko 342111, Nigeria; oluwaremilekun.ajakaye@aaua.edu.ng; 5 Department of Pure and Applied Zoology, Federal University of Agriculture, Abeokuta 110111, Nigeria; ufekpo@hotmail.com (U.F.E.); dbayegun@gmail.com (A.A.B.); 6 Department of Zoology, Akwa Ibom State University, Ikot Akpaden 520221, Nigeria; 7 AiDx Medical Bv, 3125 Schiedam, The Netherlands; tope@aidx-medical.com; 8 The Ending Neglected Diseases (END) Fund, New York, NY 10016, USA; lmakau-barasa@endfund.org (L.M.-B.); maderogba@endfund.org (M.O.A.); 9 Department of Industrial Design Engineering, Delft University of Technology, 2628 CE Delft, The Netherlands; 10 Independent Consultant, Lake Jackson, TX 77566, USA; bell00david@gmail.com; 11 CBM International, Cambridge CB5 8HY, UK; juliana.amanyi-enegela@cbm.org; 12 Department of preparatory, Brigham Young University School of Medicine, Provo, UT 84602, USA

**Keywords:** schistosomiasis, mixed infection, metagenomic sequencing, Abuja, Nigeria

## Abstract

Schistosomiasis remains a major public health concern in Nigeria. We molecularly characterized *Schistosoma* eggs obtained from human urine to identify species and assess the presence of hybrid schistosomes in Abuja, Nigeria. Urine samples were collected from 1887 participants aged five years and above. Samples were examined for Schistosoma eggs using light microscopy. A total of 507 (26.9%) were positive for any form of *Schistosoma* while 91 (4.8%) had atypical *Schistosoma* eggs. DNA extracted from pooled ova was analyzed using metagenomic sequencing, read mapping, phylogenetic analysis, and BLASTn confirmation. Molecular analyses identified genetic signatures associated with both *S. haematobium* and *S. bovis* within pooled human urine samples, indicating the co-circulation of multiple schistosome species in the study area. Phylogenetic analyses based on trans-ITS and mitochondrial COX1 markers supported the presence of distinct nuclear and mitochondrial schistosome lineages. However, because sequencing was performed on pooled egg samples, the findings cannot distinguish between true recombinants and mixed infections involving co-circulating parental species. These findings highlight the potential complexity of schistosome transmission dynamics in endemic communities and underscore the need for enhanced molecular surveillance, especially single-parasite genomic approaches, and integrated One Health investigations to better understand schistosome transmission and its implications for control and elimination efforts in Nigeria.

## 1. Introduction

Schistosomiasis is a parasitic disease caused by digenetic trematodes of the genus *Schistosoma*, transmitted to humans through freshwater snails. It remains a major public health concern, particularly in sub-Saharan Africa, where Nigeria has the highest burden. Estimates show that at least 253.7 million people required preventive treatment for schistosomiasis worldwide in 2024, with Nigeria alone accounting for approximately 134 million people at risk [1,2,3].

Five *Schistosoma* species are known to infect humans: *S. haematobium*, *S. mansoni*, *S. japonicum*, *S. intercalatum*, and *S. mekongi* [4]. Nineteen other species, inclusive of *S. bovis*, *S. mattheei*, *S. japonicum*, *S. spindale*, and *S. curassoni*, are reported to primarily infect cattle, sheep, goats, buffalo, and rodents [5]. *S. bovis*, *S. curassoni*, and *S. mattheei*, have been demonstrated among the 13,885,813 cattle, 22,092,602 sheep, and 34,453,724 goats in Nigeria, which bears the largest cattle populations in Africa, and are managed under Pastoral, Village and urban settings with low-productivity and extensive systems that face high disease risks [6,7]. However, recent studies suggest that these livestock *Schistosoma* species can hybridize with those known to infect humans, leading to zoonotic transmission. In West Africa, including Nigeria, hybrids of *S. haematobium* with *S. bovis* or *S. curassoni* have been found in humans and snails, suggesting changing transmission dynamics [8,9,10,11]. This raises concerns about misdiagnosis, treatment efficacy, and elimination efforts [12].

Abuja, Nigeria, with its mix of urban and rural settings, provides an ideal environment for human–livestock interactions. The sharing of freshwater sources by both groups may increases the risk of zoonotic schistosomiasis [13,14,15]. Reports of unusual *Schistosoma* egg shapes or hybrid schistosomiasis in human urine in Nigeria suggest the need for further investigation [16,17].

This study investigated the occurrence of hybrid schistosomes through DNA sequencing of atypical *Schistosoma* eggs found in human urine in Abuja, Nigeria.

## 2. Methods

### 2.1. Study Area and Population

**Figure 1:**
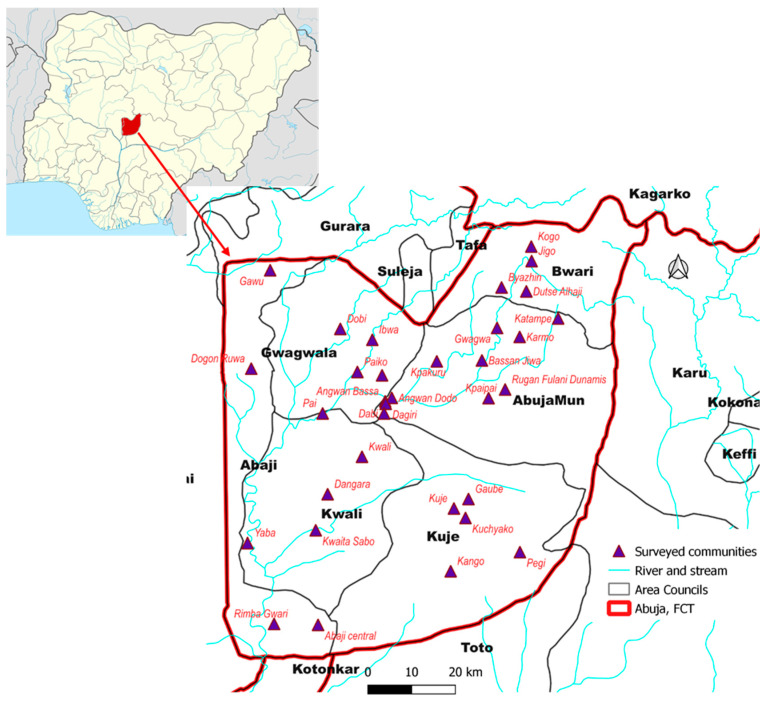
Map of Nigeria showing spatial distribution of the studied sites (produced with QGIS version 3.22.1).

This study was conducted across six Area Councils of Abuja, Nigeria (Figure 1, Supplemental Materials Table S1), between December 2022 to December 2023. Abuja is the Federal Capital of Nigeria, and a planned modern city, situated within the savannah region with moderate climatic conditions. Rainfall in the territory reflects its location on the windward side of the Jos plateau and the zone of rising air masses. The annual total rainfall is in the region of 1100–1600 mm, with wet and dry seasons. Several freshwater habitats intersect the area, some of which include ponds, streams, dams and tributaries of Gurara river stretching from Kaduna State. These water bodies form the major source of water supply to the residents of the area. During both wet and dry seasons, activities increase around these water bodies as people converge to use them for domestic, agricultural and recreational activities, all of which could predispose them to schistosomiasis [18,19]. These water bodies are shared by humans and livestock, increasing the risk of zoonotic transmission [10]. The area has a projected population of 4,026,000 as of 2024 [20], and the studied communities are shown in Figure 1.

### 2.2. Study Design and Sample Collection

The study was cross-sectional, targeting school-age children in schools and adults in the communities. A range of 50–55 subjects of both sexes was sampled from each selected community. In each community, a total of ±25 school-age children (SAC) were selected in the school using a systematic random sampling frame; ±25 adults were also selected from 25 random households at 1 participant per household from the community. A total of 1887 participants were selected for urine sample collection. Each participant received a sterile, pre-labeled 20 mL container and were instructed to void terminal urine using a standardized protocol. All samples were collected between 10:00 am and 2:00 pm after completion of consent forms and questionnaire and transported to the laboratory not later than two hours after collection. All samples were analyzed on the same day.

### 2.3. Parasitological Examination

Urine samples were gently agitated and had 10 mL withdrawn using 10 mL syringes and filtered through 13 mm polycarbonate membrane filters with 15 µm pores (Starlitech incorporation, Auburn, WA, USA) following the WHO standard protocol [21]. Filters were placed on glass slides and examined under a light microscope (with 10× objective lens and confirmed with 40× objective) for *Schistosoma* eggs. Membrane filters with unusual egg shapes (lateral spine and atypical morphology) were selected and placed in 2 mL Eppendorf tubes containing 500 µL of DNA/RNA Shield (Zymo Research, Irvine, CA, USA) to preserve nucleic acid integrity for molecular analysis. Atypical eggs were defined based on deviations from classical *S. haematobium* morphology, including altered shape and presence of non-terminal spines as described by [16].

### 2.4. DNA Extraction and Molecular Analysis

Seven membrane filters containing mixtures of both typical and atypical *Schistosoma* eggs were carefully removed from the DNA/RNA shield solution and DNA extracted using the method described by ref. [22]. The membrane filters were homogenized by bead bashing and digested by a bio-digester. The process involves adding 10 µL of proteinase k to the lysate. It was then mixed thoroughly by vortexing for 10–15 s and incubated for 1–3 h at 55 °C. To remove insoluble debris, the lysate was centrifuged at 12,000 rpm for 1 min, the aqueous supernatant was then transferred to a clean microcentrifuge tube and 1 volume of genomic binding buffer was added to the digested samples and vortexed for 15 s. The mixture was transferred to a Zymo spin column in a new collection tube and centrifuged at 12,000 rpm for 1 min. 400 µL of DNA pre-wash buffer was thereafter added to the spin column in the new collection tube and centrifuged again at 12,000 rpm for 1 min. The isolate was washed twice with g-DNA wash buffer by centrifuging at 12,000 rpm each time. The spin column was finally transferred to a clean microcentrifuge tube, 50 µL DNA elution buffer was added and incubated for 5 min at room temperature. The DNA concentration and purity were assessed using a NanoDrop ND-1000 spectrophotometer (Nanodrop Technologies LLC, Wilmington, DE, USA) [23] and integrity was confirmed on a 1.5% agarose gel electrophoresis.

The DNA amplicon from the pooled samples were subjected to a Illumina-based sequencing using transposases to simultaneously fragment and tag DNA with adapters. Library was constructed for identification by end-repair, A-tailing, and ligation of adapters followed by amplification and cleanup to remove impurities and select appropriate fragment sizes.

### 2.5. Bioinformatic Processing and Phylogenetic Analysis

Raw sequencing reads were imported into Geneious (Geneious Prime, version 2026.0). Paired-end reads were automatically associated using the default pairing settings. Read quality was assessed using the built-in quality control tools, and reads with a median Phred quality score below 20 were removed during quality filtering. Duplicate reads were identified and removed to minimize amplification bias. Reference sequences for *S. haematobium* and *S. bovis* were obtained from GenBank. Filtered reads were then mapped to the *S. haematobium* and *S. bovis* mitochondrial cytochrome oxidase 1 (COX1), and the internal transcribed spacer region (trans-ITS). Read mapping was performed using a minimum identity threshold of 95% and minimum coverage of 90%. To minimize false-positive assignments, only reads with high mapping confidence were retained. After mapping, consensus sequences were created for each gene region, and any ambiguities were noted. Variants were retained if they were supported by sufficient sequencing depth (≥15× total coverage) and by multiple reads supporting the alternate allele. For each SNP, total coverage, alternate allele read depth and frequency were calculated directly from the mapped read counts. Consensus sequences were subsequently compared against reference sequences in the NCBI GenBank database using BLASTn for species confirmation. (https://blast.ncbi.nlm.nih.gov/Blast accessed on 22 May 2023). Detailed BLAST results, including percentage identity, query coverage, and accession matches, are provided in the Supplementary Materials (Supplementary Tables S2–S4).

Consensus sequences were aligned using MUSCLE alignment in Geneious environment with relevant reference sequences obtained from GenBank and exported in FASTA format for phylogenetic analysis in MEGAX (Version 12). Phylogenetic trees for each gene dataset were inferred in MEGA X using the Maximum Likelihood (ML) method. The best-fit nucleotide substitution model for each alignment was determined using MEGA’s model selection tool based on the lowest Bayesian Information Criterion (BIC) score. ML analyses were performed under Kimura 2-parameter (trans-ITS) and Hasegawa–Kishino–Yano (COX 1) evolutionary models with 1000 bootstrap replicates to assess node support [13]. Resulting phylogenies were visualized and annotated in MEGA X, and trees were exported in PNG format. Sequence data were deposited in GenBank with accession numbers PX717347, PX717311 and PX715235 (sequence data is attached as Supplementary Materials Table S5).

## 3. Results

### 3.1. Prevalence and Distribution of Schistosoma Infection

**Figure 2:**
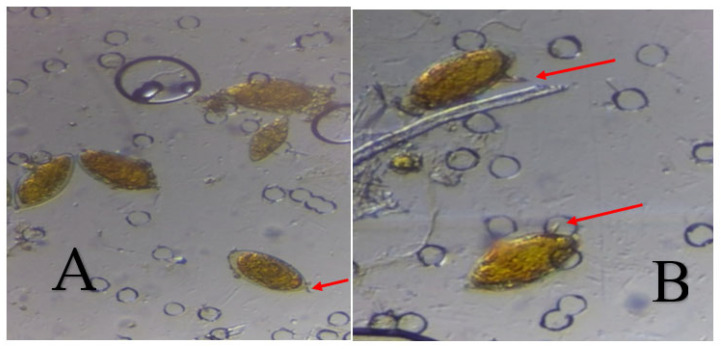
(**A**) Typical ova of S. haematobium with terminal spine (**B**) Atypical ova with lateral spine seen in urine using ×40 magnification.

The overall prevalence of urogenital schistosomiasis was 26.9%, with 4.8% of urine samples containing unusually shaped *Schistosoma* eggs (Figure 2). The Abuja Municipal Area Council recorded the highest prevalence (14.1%) of unusually shaped *Schistosoma* ova, followed by Gwagwalada (9.6%), as shown in Table 1. Similarly, 25.3%, 22% and 15.2% of those infected in the Abuja Municipal Area Council (AMAC), Gwagwalada and Kuje, respectively, had heavy intensity infection of the worms (≥50 eggs/10 mL urine) while only 9% of those infected in Abaji, Bwari and Kwali had heavy intensity infection.

**Figure 3:**
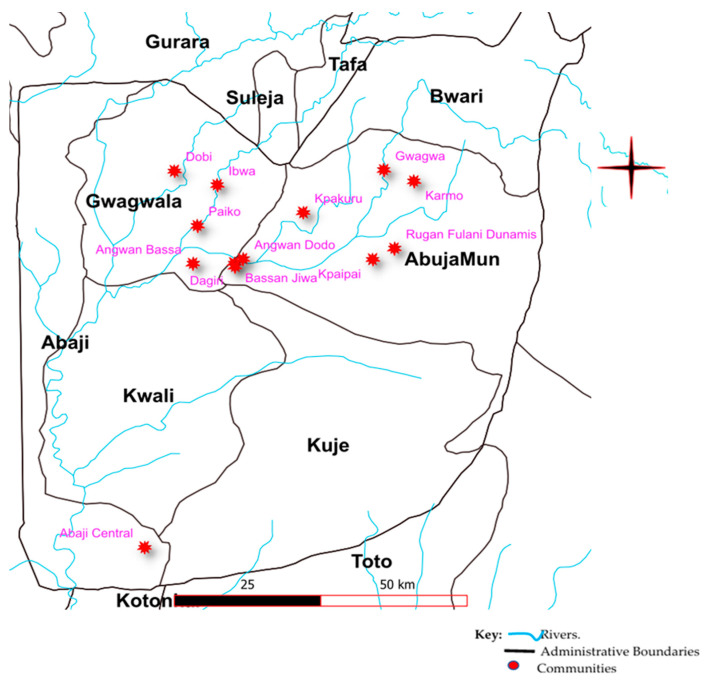
Communities where unusual *Schistosoma* ova were found in Abuja, Nigeria.(produced with QGIS version 3.22.1).

Among the 32 communities studied, 13 communities had participants with unusual *Schistosoma* eggs in their urine. The highest prevalence was recorded in Angwan Bassa (Gwagwalada Area Council), where 40% of participants had unusual ova. This was followed by Bassan Jiwa in (AMAC) with 30% and Gwagwa in (AMAC) with 26% (Supplemental Materials Table S6). A geospatial analysis of these communities revealed that these communities with high incidence of unusual schistosoma ova are located near multiple intersecting rivers and water bodies (Figure 3).

### 3.2. Genetic Profiling of Schistosoma Species

Both the *S. haematobium* and *S. bovis* mitochondrial and nuclear *S. haematobium* genotypes were successfully obtained from the pooled sample (details in Supplementary Materials Table S5). BLASTn analysis confirmed the presence of *S. haematobium* and *S. bovis*-like sequences, with top hits showing 98–100% nucleotide identity to GenBank reference sequences (Supplementary Materials Tables S2–S4). Due to the conserved nature of the trans-ITS, the reads were mapped against the *S. haematobium* reference only and variations across the loci were identified. A total of seven single nucleotide polymorphisms (SNPs) were identified across the *trans-ITS* (Table 2). Total sequencing coverage at variant positions ranged from 41× to 80×, with alternate allele read depths between 12 and 22 reads. Alternate allele frequencies ranged from 21.3% to 38.8%, indicating substantial population-level variation across the trans-ITS. Given the pooled metagenomic design, these SNP frequencies should be interpreted as population-level signals and not as direct evidence of within-individual heterozygosity. Four of the variant positions represented SNP positions between *S. haematobium* and *S. bovis* at the *ITS2* while the rest were private SNPs.

### 3.3. Phylogenetic Analysis

**Figure 4:**
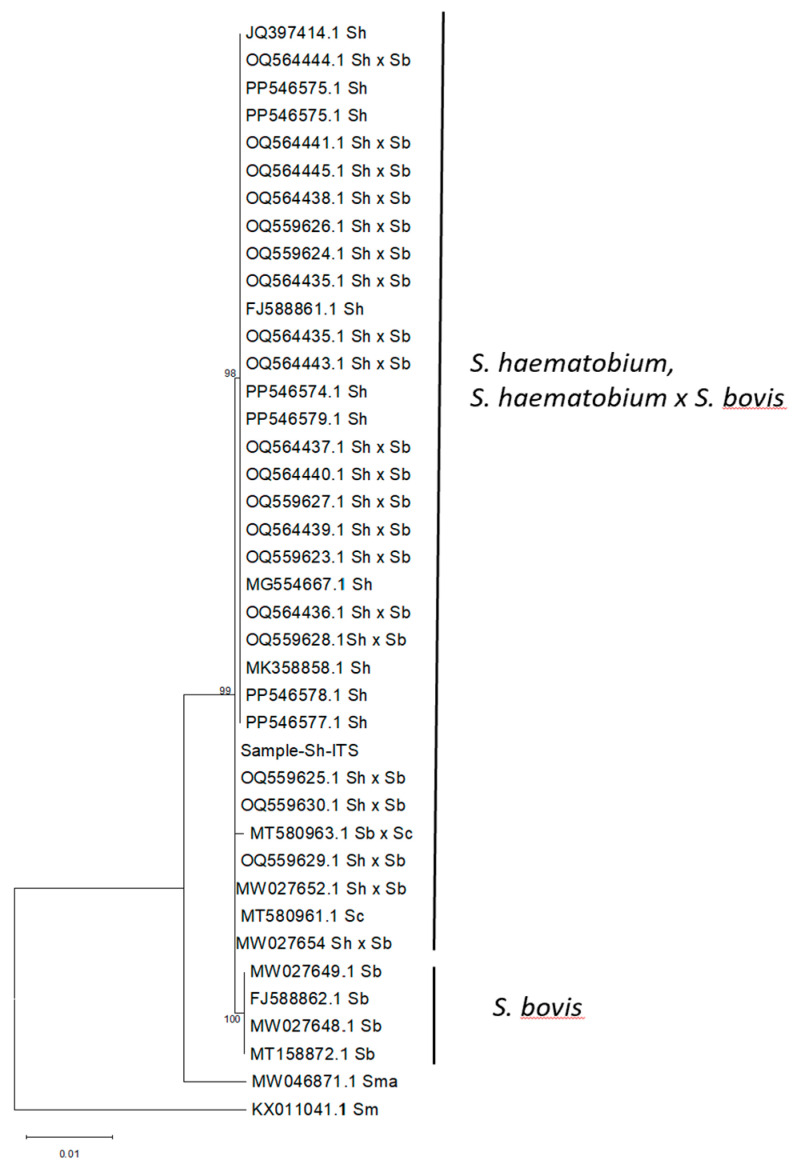
Maximum Likelihood phylogenetic tree based on trans-ITS sequences of *Schistosoma* species from Africa. Reference sequences comprise published sequences retrieved from GenBank with *S*. *mansoni* (KX011041) used as the outgroup. (Sample-Sh-ITS—this study; *S*h = *S*. *haematobium*; Sb = *S*. *bovis*; *S*h *×* Sb = *S*. *haematobium* × *S*. *bovis*; Sc = *S*. *curassoni*; Sb x Sc = *S*. *bovis* × *S*. *curassoni*; Sma = *S*. *mattheei Sm* = *S*. *mansoni*.)

The nuclear trans-ITS phylogenetic analysis was inferred from the single DNA sequence (875 bp) and selected *S. haematobium* and *S. bovis* sequences from Nigeria and other parts of Africa obtained from NCBI using the Maximum Likelihood method in MEGA X. The generated tree showed strong bootstrap support across major nodes (98–100%) with the sample *S. heamatobium* sequence (Sample-Sh-ITS) clustering within the *S. haematobium* and *S. haematobium* × *S. bovis* lineages. These lineages were clearly separated from the Sb/Sma/Sm lineages, which formed a distinct, strongly supported clade (Figure 4).

**Figure 5:**
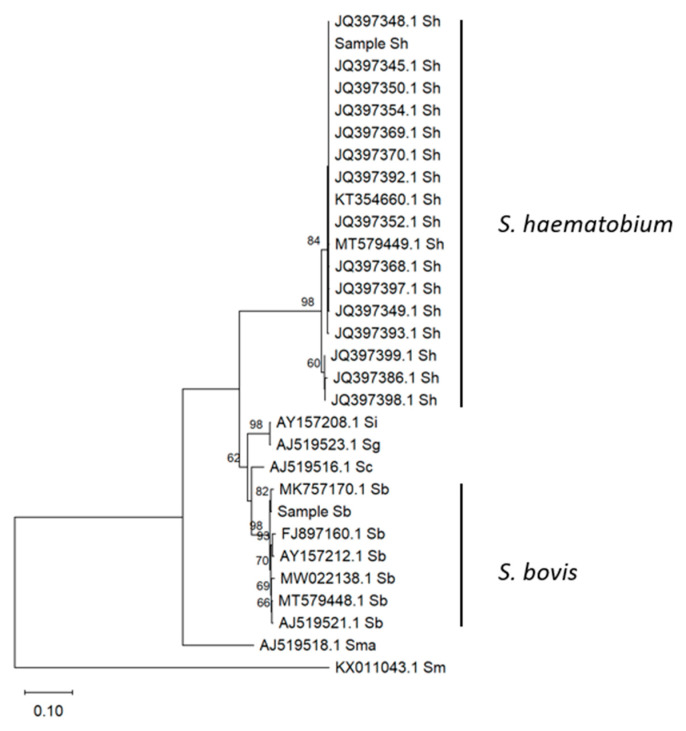
Maximum Likelihood phylogenetic tree based on COX 1 sequences of *Schistosoma* species from Africa. Reference sequences comprise published sequences retrieved from GenBank with *S*. *mansoni* (KX011043) used as the outgroup. (Sample Sh and Sample Sb—this study; *S*h = *S*. *haematobium*; Sb = *S*. *bovis*; Sc = *S*. *curassoni*; *Sg* = *S. guineensis*, Sma = *S*. *mattheei Sm* = *S*. *mansoni*.)

The mitochondrial COX1 phylogeny demonstrated distinct clustering patterns corresponding to *S. haematobium* and *S. bovis* reference sequences. The study-derived sequences (953bp) were grouped within their respective species-associated mitochondrial clades, supporting the presence of mitochondrial haplotypes related to both species within the analyzed pooled sample (Figure 5).

## 4. Discussion

This study provides molecular evidence indicating the presence of both *S.haematobium* and *S. bovis* genetic signatures in human urine samples from Abuja, Nigeria. These findings suggest the co-circulation of multiple schistosome species within endemic communities and raise important questions regarding transmission dynamics, including the potential for zoonotic interactions.

The detection of *S. bovis*-associated mitochondrial sequences alongside *S. haematobium* signatures is consistent with previous reports from West Africa demonstrating introgressive hybridization and overlapping transmission cycles between human and livestock schistosomes [9,14,24,25]. However, it is important to interpret these findings cautiously. Because DNA was extracted from pooled egg samples, it is not possible to distinguish whether the observed genetic patterns reflect true hybrid parasites or mixed infections involving multiple species across individuals. As such, the present data support the presence of multi-species transmission but do not provide definitive evidence of hybridization at the individual parasite level.

Microscopic examination revealed the presence of atypical eggs, including forms with lateral spines. The presence of these eggs with a typical lateral spine in human urine is highly unusual, as neither *S. haematobium* nor *S. bovis* normally possesses this characteristic except *S. mansoni* [16]. These eggs with a lateral spine may have result from a more complex hybridization involving *S. mansoni* as such morphological variation has been previously associated with hybrid schistosomes. However, egg morphology alone is an unreliable indicator of genetic ancestry [26]. In the absence of direct molecular characterization of individual eggs, the relationship between atypical morphology and underlying genotype remains speculative.

The identification of *S. bovis* genetic material in human-derived samples highlights the potential role of livestock in sustaining transmission cycles. In settings such as Abuja, where humans and animals frequently share freshwater contact sites, opportunities for cross-species transmission may be substantial [27,28,29]. Nonetheless, this study did not include sampling of animal hosts or intermediate snail populations, and therefore cannot directly confirm zoonotic transmission. Future investigations incorporating parallel sampling of humans, livestock, and snail hosts will be essential to elucidate the directionality and ecological drivers of transmission.

Genetic analysis of the trans-ITS region revealed multiple single nucleotide polymorphisms, including positions consistent with divergence between *S. haematobium* and *S. bovis* [14,30]. The presence of intermediate allele frequencies across several loci suggests genetic heterogeneity within the sampled population.

The COX1 phylogenetic analysis provided additional mitochondrial evidence supporting the genetic relationship of the detected sequences with *S. haematobium* and *S. bovis*. Unlike the highly conserved 18S rRNA marker, mitochondrial COX1 possesses greater discriminatory power for resolving closely related schistosome taxa and has been widely used in studies investigating introgression and hybridization within the *S. haematobium* species complex [9,14]. The concordance between the nuclear trans-ITS and mitochondrial COX1 analyses strengthens the interpretation that multiple schistosome genetic backgrounds are present within the pooled sample. However, because the present study was based on pooled eggs rather than single-worm or single-egg genotyping, the phylogenetic patterns observed cannot definitively distinguish recombinants from mixed infections involving co-circulating parental species. Consequently, the findings should be interpreted as evidence of population-level genetic admixture rather than conclusive proof of hybridization. Furthermore, the present dataset does not permit discrimination between recent hybridization events and ancient introgression, as similar discordant patterns between nuclear and mitochondrial markers may persist following historical backcrossing and admixture. Recent genomic studies have demonstrated that ITS and mitochondrial markers alone may overestimate contemporary hybridization and may instead reflect older introgressed lineages within endemic schistosome populations [31]. Additional multilocus or whole-genome analyses using single egg genotyping would be required to accurately characterize population structure and determine the extent and directionality of introgression between *S. haematobium* and *S. bovis* lineages.

This study has several important limitations. Most notably, the pooling of eggs from multiple individuals prior to DNA extraction prevents attribution of genetic signatures to specific hosts or egg morphologies. This limits the ability to distinguish between mixed species infection and hybridization and precludes individual-level inference. Additionally, the absence of animal and snail sampling restricts conclusions regarding transmission pathways. Finally, the reliance on targeted genetic markers rather than whole-genome data constrains the resolution of species identification and hybrid detection.

Despite these limitations, this study provides valuable preliminary molecular data suggesting the presence of livestock-associated schistosome genetic material in human populations in Nigeria. These findings underscore the need for enhanced molecular surveillance and more integrative study designs to better understand schistosome transmission dynamics in endemic settings.

## Figures and Tables

**Table 1:** Prevalence and intensity of Schistosoma ova in human urine by Local Area Councils.

Local Area Councils	No. Tested	No. Positive for any *Schistosoma* Eggs (%)	No. Positive for Atypical *Schistosoma* Eggs	% (95% Confidence Interval) for Atypical *Schistosoma*	Overall Intensity Classification (WHO)	
					Light: 1–49 Eggs/10 mL Urine (%)	Heavy: ≥50 Eggs/10 mL Urine (%)
Abaji	247	64 (25.9)	4	1.6 (0.04–3.2)	58 (23.5)	6 (2.4)
AMAC	306	150 (49)	43	14.1 (10.2–17.9)	112 (36.6)	38 (12.4)
Bwari	358	22 (6.1)	0	0 (0.0–0.84)	20 (5.6)	2 (0.6)
Gwagwalada	459	141 (30.7)	44	9.6 (6.9–12.3)	110 (24)	31 (6.8)
Kuje	252	66 (26.2)	0	0 (0.0–1.19)	56 (22.2)	10 (4.0)
Kwali	265	64 (24.2)	0	0 (0.0–1.13)	58 (21.9)	6 (2.3)
Total	1887	507 (26.9)	91	4.8 (3.9–5.8)	414 (21.9)	93 (5.0)

**Table 2:** SNPs at the trans-ITS identifed in pooled sample.

Position	Ref	Alt	Coverage	Variant Depth	Variant Frequency (%)
576	C	T	46	12	26.1
745	G	A	49	19	38.8
800	C	T	47	18	38.3
850	G	A	41	15	36.6
920	C	T	47	14	29.8
977	G	A	76	22	28.9
978	G	A	80	17	21.3

## Data Availability

All sequences generated in this study have been deposited in the NCBI DNA Data Bank and accession numbers issued. The accession numbers are: PX717347, PX717311 and PX715235 for consensus sequences and PRJNA1353939 for raw reads.

## Supplementary Materials

The following supporting information can be downloaded at: https://www.mdpi.com/article/10.3390/tropicalmed11060170/s1, Table S1: Surveyed communities and their geo-coordinates; Table S2: NCBI Nucleotide Blast Result for Nuclear transITS Sequence, *S. haematobium*; Table S3: NCBI Nucleotide Blast Result for Mito-chondrial COX 1 Sequence, *S. haematobium*; Table S4: NCBI Nucleotide Blast Result for Mitochon-drial COX 1 Sequence, *S. bovis*; Table S5: The sequence information which were deposited in Gen-Bank; Table S6: prevalence of unusual Schistosoma ova in urine by Community.
